# Skeletal Muscle Mass and Higher-Level Functional Capacity in Female Community-Dwelling Older Adults

**DOI:** 10.3390/ijerph18136692

**Published:** 2021-06-22

**Authors:** Shuichi Wakayama, Yoshihiko Fujita, Keisuke Fujii, Takeshi Sasaki, Hiroshi Yuine, Kazushi Hotta

**Affiliations:** 1 Department of Occupational Therapy, Ibaraki Prefectural University of Health Sciences, 4669-2 Ami-Machi, Inashiki-gun, Ibaraki 300-0394, Japan; fujitay@ipu.ac.jp (Y.F.); sasakita@ipu.ac.jp (T.S.); yuinehi@ipu.ac.jp (H.Y.); hotta@ipu.ac.jp (K.H.); 2 Department of Occupational Therapy, Kansai University of Health Sciences, Kumatoricho, Sennan-gun, Osaka 590-0482, Japan; k.fujii@kansai.ac.jp

**Keywords:** skeletal muscle mass, higher-level functional capacity, elderly community residents

## Abstract

Purpose: In this study, our purpose was to examine the relationship between skeletal muscle mass and higher-level functional capacity in female community-dwelling older adults. Participant(s) and Methods: In this cross-sectional study, we targeted 55 female community-dwelling older adults aged 65 years and above participating in long-term care prevention classes in Ibaraki Prefecture between 2018 and 2020. We excluded individuals with cognitive impairment and those judged as having sarcopenia. The variables of interest included age, height, weight, body mass index, skeletal muscle mass index (SMI), handgrip strength, step count, and family structure. We calculated the SMI by dividing the extremities’ total lean mass by the square of the height (in m), while the number of steps was calculated using the three-axis accelerometer Actigraph GT3X^®^. We measured skeletal muscle mass via bioelectrical impedance analysis using the InBody270 body composition analyzer and muscular strength as grip strength. Results: We observed significant relationships between skeletal muscle mass and Tokyo Metropolitan Institute of Gerontology Index of Competence (TMIG-IC) (β = 0.336, *p* < 0.01) and handgrip strength (β = 0.230). Conclusion: In this study, a relationship between skeletal muscle mass and higher-level functional capacity was demonstrated among elderly female community residents.

## 1. Introduction

The prevention of sarcopenia is critical in care prevention and health promotion among older people in Japan. In a 2014 study by Chen et al., sarcopenia was identified based on three factors: loss of skeletal muscle mass, decreased muscular strength and decreased motor function [1]. However, the Asian Working Group for Sarcopenia (AGWS) revised the diagnostic criteria for the condition in 2019 [2]. This revision was carried out to detect and prevent sarcopenia in high-risk older adults without the need for measuring skeletal muscle mass using technical equipment. Additionally, in 2013, Malmstrom and Morley recommended the use of the Strength, Assistance with Walking, Rise from a Chair, Climb Stairs, Falls questionnaire (SARC-F) [3], which determines the presence of sarcopenia based on items related to activities of daily living (ADL). Previous reports have indicated that sarcopenia is strongly associated with low levels of ADL functionality and higher-level functional capacity [4] and that it has a significant impact on daily life.

Further, reports have detailed concerns that older adults who are not categorized as having sarcopenia but have low skeletal muscle mass experience impaired instrumental activities of daily living (IADL) capacity. Wang et al. have shown that in elderly individuals, skeletal muscle mass is a predictive factor for reduced IADL capacity [5]. The European Working Group on Sarcopenia in Older People (EWGSOP) considers the state of having skeletal muscle mass below the standard level in the absence of other criteria for sarcopenia, such as reduced muscular strength and walking speed, as pre-sarcopenia [6]. Bianchi et al. showed that among 483 local elderly residents, 70.9% of those with sarcopenia exhibited impaired IADL capacity, as did 24.5% of those with low skeletal muscle mass. Elderly individuals without sarcopenia essentially maintain ADL capacity [7]. Nonetheless, it seems that reduced higher-level functional capacity, including IADL capacity, plays a role in skeletal muscle mass loss. Koyano et al. divide higher-level functional capacity into three elements: IADL, intellectual activity, and social role [8].

Amidst societal aging in Japan, to enable elderly individuals to lead independent lives, it is critically important to find ways to help them to maintain their higher-level functional capacity. However, few previous reports have examined the relationship between skeletal muscle mass and higher-level functional capacity in community-dwelling older adults who do not have sarcopenia, which, we believe, is important from the perspective of sarcopenia prevention. We must enable individuals to maintain and improve their functional capacities. If we can clarify the nature of the relationship between skeletal muscle mass and higher-level functional capacity, it would serve as an essential reference for interventional methods for sarcopenia prevention. Thus, this study examines the relationship between skeletal muscle mass and higher-level functional capacity among community-dwelling older adults.

## 2. Materials and Methods

### 2.1. Participants

In this cross-sectional study, participants were recruited from long-term care prevention classes held in City A in Ibaraki Prefecture between 2018 and 2020. The study was advertised in the classroom through a PR magazine. The target area, City A in Ibaraki Prefecture, is a rural area located on the Kanto Plain east of Japan and in the southern part of Ibaraki Prefecture. As of April 2021, the total population was 48,012, and the aging rate was 28.3%, comparable to Japan’s overall aging rate of 28.4%. The inclusion criteria were female older adults aged 65 years and above. Exclusion criteria were the presence of cognitive impairment or sarcopenia. In this manner, of the 55 females who were recruited, 4 were excluded. We carried out sarcopenia diagnoses per the Asian Working Group for Sarcopenia 2019 criteria: (1) skeletal muscle mass index (SMI) less than 5.7 kg/m^2^, as measured by bioelectrical impedance analysis and (2) low muscular strength (handgrip strength less than 18 kg) and/or low physical functionality (walking speed less than 1.0 m/s or a modified five times sit-to-stand test (SS-5) measurement of 12 or less).

### 2.2. Measurement Variables

The variables of interest were age, height, weight, body mass index (BMI), Fat Mass Index (FMI), SMI, handgrip strength, and family structure. Skeletal muscle mass was measured via bioelectrical impedance analysis using the InBody270 body composition analyzer (InBody Japan). Body composition analysis was performed for all subjects at the same time and date. Each subject had breakfast at home, did not have any exercise in advance, and participated in a brief orientation. The measurement time per person was about 2.3 min, and measurements were taken from 10 to 12 a.m. We calculated the SMI by dividing the extremities’ total lean mass by the square of the height (in m). The number of steps was calculated using the three-axis accelerometer Actigraph GT3X^®^ (Actigraph).

We used grip strength as a measure of overall muscular strength. The grip strength meter was held at the body’s side in a standing position, gripped with maximum effort. Physical functions were measured using handgrip strength, SS-5, walking speed, and step count. For the SS-5, we measured the time it took for participants to stand up from a seated position in a chair and then sit down again a total of five times. For walking speed, we measured the time it took participants to walk down a 5 m path.

The Tokyo Metropolitan Institute of Gerontology Index of Competence (TMIG-IC) was used to survey higher-level functional capacity [8]. The 13-item TMIG-IC consists of three subscales—IADL (five items), intellectual activity (four items), and social role (four items)—with a maximum score of 13 points. IADL comprises five components: going out using public transportation, shopping for daily necessities, preparing meals, paying bills, and withdrawing deposits and savings. Intellectual activity comprises filling out forms, reading newspapers, reading books, and interest in health. Social role comprises visiting friends at their homes, consulting with family and friends, visiting sick friends, and talking with young people. The higher the score, the higher the individual’s functionality.

### 2.3. Statistical Analysis

For our statistical analyses, we used a partial correlation test to examine the relationships between skeletal muscle mass and each of the following: muscular strength, physical function, and higher-level functional capacity. Furthermore, to clarify the relationship between skeletal muscle mass and higher-level functional capacity, we performed a multiple regression analysis with skeletal muscle mass as the dependent variable. In Model 1, grip strength and overall TMIG-IC score were input as explanatory variables. Moreover, to clarify the relationship between skeletal muscle mass, TMIG-IC subscale scores were input into Models 2 as explanatory variables. The covariates of age and FMI were introduced in the partial correlation test and all models of multiple regression analysis. The independent input variable confirmed multicollinearity with a correlation matrix; thus, we set the correlation coefficient to less than 0.7. Statistical analyses were performed with SPSS Statistics 24.0 (IBM Corp., Armonk, NY, USA), and the significance level for all tests was set at 5%.

### 2.4. Ethical Considerations

We explained this study’s purpose and the confidentiality obligation regarding personal information to the participants and obtained oral and written consent from them before participation. We conducted this study with the Ibaraki Prefectural University of Health Sciences Research Ethics Committee’s approval (approval number: e212).

## 3. Results

The basic characteristics of the 51 individuals that participated in this study are as follows: Their mean age was 73.5 ± 4.8 years, mean SMI was 6.0 ± 0.7 kg/m^2^, mean step count per day was 12283 ± 4329 steps, and 11 individuals (21.7%) lived alone. Mean handgrip strength was 22.4 ± 3.2 kg, and in terms of physical functionality, mean SS-5 time was 6.8 ± 2.0 s and mean walking speed was 1.4 ± 0.2 m/s. Mean TMIG-IC score, an indicator of higher-level functional capacity, was 12.4 ± 0.8, IADL score was 4.8 ± 0.5, intellectual activity score was 3.7 ± 0.6, and mean social role score was 3.8 ± 0.4 (Table 1).

### 3.1. Comparison of Skeletal Muscle Mass with Muscular Strength, Physical Functionality, and Higher-Level Functional Capacity

The correlation of skeletal muscle mass with muscular strength, physical functionality, and higher-level functional capacity is demonstrated in Table 2. We observed significant correlations between skeletal muscle mass and handgrip strength (r = 0.290), SS-5 (r = −0.317), TMIG-IC score (r = 0.448), IADL score (r = 0.349), and social role score (r = 0.294). However, skeletal muscle mass displayed no significant correlation with walking speed and intellectual activity.

### 3.2. Multiple Regression Analysis with Skeletal Muscle Mass as the Dependent Variable

In the model in which we set skeletal muscle mass as the dependent variable, handgrip strength and overall TMIG-IC score were the explanatory variables (model 1, Table 3). We observed significant relationships between skeletal muscle mass and TMIG-IC (β = 0.336, *p* < 0.01) and handgrip strength (β = 0.230, *p* < 0.05). The adjusted R^2^ was 0.507.

To clarify the relationship between skeletal muscle mass and TMIG-IC subscales, we constructed a model in which we set skeletal muscle mass as the dependent variable and handgrip strength and three subscales as explanatory variables (model 2). There were significant observable relationships between skeletal muscle mass and handgrip strength (β = 0.203, *p* < 0.05) and IADL score (β = 0.187, *p* < 0.05). The adjusted R^2^ was 0.689.

To clarify the relationship between skeletal muscle mass and social role, we constructed a model in which skeletal muscle mass was the dependent variable and handgrip strength and social role were explanatory variables (model 3). There were significant observable relationships between skeletal muscle mass and IADL score (β = 0.236, *p* < 0.05), social role (β = 0.232, *p* < 0.05), and handgrip strength (β = 0.229, *p* < 0.05). The adjusted R^2^ was 0.501.

## 4. Discussion

Because skeletal muscle mass distributions vary by race and ethnicity, we compared these values to the National Health and Nutrition Survey, which targets Japanese citizens [9]; mean SMI values by age in this survey were 6.5 kg/m^2^ for females between 65 and 74 years and 6.2 kg/m^2^ for those older than 75. A total of 51 elderly female community residents participated in this study; their mean age was 73.5 ± 4.8 years, and their mean SMI was 6.0 ± 0.7 kg/m^2^. Their mean step count was 12283 ± 4329 steps; compared to the mean step count of elderly community residents published by the Ministry of Health, Labor, and Welfare [9], this group’s activity level was high. Further, in terms of their physical functionality, their grip strength was 22.4 ± 3.2 kg, their mean SS-5 time was 6.8 ± 2.0 s, and their walking speed was 1.4 ± 0.2 m/s. Thus, while we did not classify the participants surveyed in this study as having sarcopenia, they are certainly a low-SMI group. Further, according to a physical fitness and athletic ability survey carried out by the Japan Sports agency [10], female older adults aged between 70 and 74 have a mean grip strength of 23.9 ± 3.9 kg, and those aged between 75 and 79 have a mean grip strength of 22.6 ± 3.9 kg. Thus, our participants exhibited slightly low values for their age group. Concerning SS-5 and walking speed, compared to previous studies of community-dwelling older adults [11,12], our participants exhibited good values, perhaps because of their involvement in a municipal care prevention class. From their mean daily step count, we can state that they were a relatively active group. Further, while these individuals were not sarcopenic, their SMI was low, their grip strength was slightly low, and their physical functionality was good. Yamada et al. reported the morbidity of these three items in older adults aged above 65 in 10-year groups, finding that skeletal muscle mass, muscular strength, and physical functionality were highest in that order [13]. In this study, too, it is possible that as these individuals age, their skeletal muscle mass will fall, which will lead to reduced muscular strength, and then to reduced physical functionality, ultimately engendering sarcopenia.

### 4.1. The Association between Skeletal Muscle Mass and Muscular Strength/Physical Functionality

In terms of physical functionality, skeletal muscle mass was associated with grip strength, but not walking speed. There are previous observable relationships with several items in terms of the correlation between skeletal muscle mass and physical functionality, including grip strength [14,15]. As explained previously, this study’s participants had low SMI and slightly low grip strength, but their physical functionality was good. Similarly, in a report by Mori et al., while pre-sarcopenic elderly individuals had significantly lower grip strength than elderly individuals with normal skeletal muscle mass, no significant difference was observed for walking speed [16]. In this report, the authors speculate that even if a slight loss of muscular strength occurs due to a decline in muscle mass, one’s remaining reserve capacity is enough to maintain walking speed. As some of the study participants were pre-sarcopenic, we believe our results demonstrate the same principle. Further, in Auyeung et al.’s longitudinal survey of age-related changes in muscular strength, grip strength, and walking speed in healthy community-dwelling older adults, the baseline age, skeletal muscle mass, and grip strength in female participants were the same as those of our participants [17]. That report states that while the loss of skeletal muscle mass observed in female older adults over the course of four years was slight, the loss of grip strength over the course of just two years was considerable. This finding suggests that grip strength decreases relatively quickly with age. We believe that to prevent skeletal muscle mass loss, early efforts to improve muscular strength are necessary.

### 4.2. The Relationship between Skeletal Muscle Mass and Higher-Level Functional Capacity

Among measures of higher-level functional capacity, the variables that correlated significantly with skeletal muscle mass were TMIG-IC (r = 0.448), IADL (r = 0.349), and social role (r = 0.294). One of the subscales of TMIG-IC, intellectual activity, did not correlate significantly with skeletal muscle mass. To clarify the relationship between skeletal muscle mass and these various factors, we performed a multiple regression analysis. In a model in which we set skeletal muscle mass as the dependent variable and input grip strength and overall TMIG-IC score as explanatory variables (Table 3 model 1), skeletal muscle mass exhibited significant correlations with TMIG-IC score (β = 0.336, *p* < 0.01) and grip strength (β = 0.230, *p* < 0.05). In a model with skeletal muscle mass as the dependent variable, and grip strength and three subscales input as explanatory variables (Table 3 model 2), skeletal muscle mass demonstrated significant correlations with IADL score (β = 0.236, *p* < 0.05), social role (β = 0.232, *p* < 0.05), and grip strength (β = 0.229, *p* < 0.05). Regarding the existing literature, Tanimoto et al. reported an association between sarcopenia and TMIG-IC subscale values in female community-dwelling older adults [4]. However, Momoki et al. did not observe anything of significance when examining TMIG-IC scores as factors associated with sarcopenia in elderly female community residents [18]. Momoki et al. note that they used the median score as the TMIG cutoff and comment that this may have been a slightly high threshold. The mean TMIG-IC score in this study was 12.4 ± 0.8 points, a relatively high value, but it was a continuous variable in multiple regression analysis. These findings suggest that a decrease in skeletal muscle mass may slightly reduce higher-level functional capacity, and this requires further investigation.

One of our study’s characteristics is our investigation of the correlation of higher-level functional capacity subscales with skeletal muscle mass. Here, we constructed three models with skeletal muscle mass as the dependent variable: one with an overall TMIG-IC score as an explanatory variable, one with an IADL score, and one with the social role (Table 3). All three of these subscale factors—TMIG-IC, IADL, social role—were correlated with skeletal muscle mass, even after adjusting age, BMI, and muscular strength. However, intellectual activity, which is one of the subscales of TMIG-IC, was not significantly associated with skeletal muscle mass in a single correlation analysis. Just as IADL is considered to be a factor associated with sarcopenia in terms of its correlation with skeletal muscle mass [5], our results suggest that IADL level may be associated with loss of skeletal muscle mass even if decreased muscular strength and/or physical functionality is not observed. Intellectual activity corresponds to Lowton’s model of “situational responsiveness” and is active, including leisure, learning activities, and creativity [19]. While intellectual activity and the social role both involve daily activities, in particular, intellectual activity consists of things such as “filling out pension forms”, “reading the newspaper, books, or magazines”, and “interest in health-related programs and articles”. As a result, it does not include elements that require physical activity and is therefore unlikely to be affected by differences in skeletal muscle mass. Social role is believed to be a functional evaluation index for old age [20] and has been pointed out to perhaps decrease faster than IADL and intellectual activity [21]. Social role is also believed to involve daily habits. Nonetheless, because it includes tasks such as “visiting a friend’s house” or “seeing a friend in the hospital”, which require physical movement, we believe it is valid that we observed it to be correlated with skeletal muscle mass. Thus, preventing a decrease in skeletal muscle mass may be useful in promoting social activities.

In this study, we have identified a relationship between skeletal muscle mass and higher-level functional capacity. In particular, we have demonstrated correlations between skeletal muscle mass and IADL and social role, both of which involve physical elements. These findings suggest that support for IADL level/social role, both of which are higher-level functions, is necessary to preserve and maintain skeletal muscle mass as a way to prevent sarcopenia, in addition to early interventions designed to maintain and improve muscular strength. While it has been reported that compared to males, females exhibit a more gradual age-related decline in skeletal muscle mass [22], one factor influencing this result may be that in Japan, females, rather than males, are more frequently involved in housework and social activities. On the other hand, it has been reported that the prevalence of sarcopenia is higher in older females than in older males, and that low-relative skeletal muscle mass is associated with impairment and physical disability [23]. Although this study only targeted older females, it is expected to support IADL and social activities to prevent the occurrence of sarcopenia in older females.

### 4.3. Limitations of This Study

The relationship between sarcopenia and higher-level functional capacity has been previously reported. In this study, the relationship between skeletal muscle mass and higher-level functional capacity was observed even in older adults who were not sarcopenic. However, this study employed a cross-sectional design, and a longitudinal observation is essential to clarify the causal relationship present here. This study’s participants were healthy older females who participated in a care prevention class conducted in the community and did not represent a randomly selected sample. Most of the participants in our class were females, so the study did not include older males. Therefore, it is difficult to generalize this study to patients with diseases or to older male participants. In the future, it will be necessary to conduct a randomized study targeting a wide range of older adults living in the community. Additionally, the higher-level functional capacity of our participants was relatively high. We observed the relationships between skeletal muscle mass and IADL/social role. It is crucial to carry out more detailed investigations of the association between higher-level functional capacity and sarcopenia to prevent the decrease in skeletal muscle mass as a means of achieving early prevention of sarcopenia.

## 5. Conclusions

In this study, a relationship between skeletal muscle mass and higher-level functional capacity was shown among elderly female community-dwelling older adults. In particular, among the higher-level functional capacity components, relationships between IADL level and social role were indicated. These findings suggest that, in addition to early interventions designed to maintain and improve muscular strength, support for higher-level functions is necessary to preserve and maintain skeletal muscle mass as a way to prevent sarcopenia.

## Figures and Tables

**Table 1:** Characteristics of the participants.

Variable	Value
Age (years)	73.5 ± 4.8
Height (cm)	152.6 ± 6.0
Weight (kg)	53.8 ± 8.0
BMI (kg/m^2^)	23.1 ± 3.0
FMI (kg/m^2^)	7.4 ± 2.1
SMI (kg/m^2^)	6.0 ± 0.7
Living alone (%)	11(21.7)
Muscular strength	
Hand grip strength (kg)	22.4 ± 3.2
Physical function	
SS-5 (s)	6.8 ± 2.0
Walking speed (m/s)	1.4 ± 0.2
Steps count (steps/day)	12,283 ± 4329
Higher-level functional capacity	
TMIG-IC score (point)	12.4 ± 0.8
IADL (point)	4.8 ± 0.5
Intellectual activity (point)	3.7 ± 0.6
Social role (point)	3.8 ± 0.4

Values are mean ± standard deviation or presented as *n* (%); BMI: body mass index; FMI: Fat Mass Index; SMI: skeletal muscle mass index; SS-5: Sit to Stand-5; TMIG-IC: Tokyo metropolitan Institute of Gerontology Index Competence; IADL: Instrumental activities of daily living.

**Table 2:** Relationships between skeletal muscle mass and each of the following: muscular strength, physical function, and higher-level functional capacity.

Variable	r
Muscular strength	
Hand grip strength (kg)	0.290 *
Physical function	
SS-5 (s)	−0.317 *
Walking speed (m/s)	0.124
Steps Count (steps/count)	0.015
Higher-level functional capacity	
TMIG-IC score (point)	0.448 **
IADL (point)	0.349 **
Intellectual activity (point)	0.166
Social role (point)	0.294 *

Partial correlation test * *p* < 0.05, ** *p* < 0.01; Covariates of age and FMI were introduced.

**Table 3:** Multiple regression analysis with skeletal muscle mass as the dependent variable.

	Model 1		Model 2	
	β	95% CI	β	95% CI
Hand grip strength	0.230 *	0.004–0.093	0.229 *	0.003–0.093
TMIG-IC score	0.336 **	0.102–0.403		
IADL			0.236 *	0.034–0.608
Intellectual activity			0.111	−0.117–0.386
Social role			0.232 *	0.038–0.635

Both models were adjusted for age and FMI. 95% CI: Confidence interval * *p* < 0.05, ** *p* < 0.01. R^2^/Adjusted R^2^ of model 1: 0.546/0.507, R^2^/Adjusted R^2^ of model 2: 0.560/0.501.

## Data Availability

Data not available due to ethical restrictions.
